# Supplementary material for: Highly efficient and salt rejecting solar evaporation via a wick-free confined water layer
Source: Nat Commun. 2022 Feb 14;13:849. doi: 10.1038/s41467-022-28457-8 (PMC8844429; doi:10.1038/s41467-022-28457-8)
Supplement: Supplementary file 1 — Supplementary Information [file 41467_2022_28457_MOESM1_ESM.pdf]

Supplementary Information

Highly efficient and salt rejecting solar evaporation via a wick-free confined water layer

Lenan Zhang[1,†], Xiangyu Li[1,†], Yang Zhong[1], Arny Leroy[1], Zhenyuan Xu[2], Lin Zhao[1], Evelyn N. Wang[1,*]

[1]Department of Mechanical Engineering, Massachusetts Institute of Technology, Cambridge, MA 02139, USA

[2]Institute of Refrigeration and Cryogenics Shanghai Jiao Tong University, Shanghai 200240, China

[†]Equal contribution to this work

[*]Corresponding author: enwang@mit.edu (E.N. Wang)

Supplementary Note 1: Device design and fabrication

To enable simultaneous thermal localization and salt rejection, we optimized the water macrochannel size and insulation thickness, as shown in Supplementary Figure 1. The heat and salt transport through a single-channel unit was firstly simulated to obtain the key design parameters. Temperature and salinity profiles in the second hour are shown with different macrochannel sizes (1 mm to 5 mm) and insulation thicknesses (6.4 mm to 38.1 mm). When the macrochannel diameter was smaller than 2 mm, the natural convection was weak, leading to a highly concentrated brine in the confined water layer. When the macrochannel diameter was larger than 4 mm, although no salt accumulation can be observed, heat loss through the macrochannel was significant due to the strong natural convection. Similar design trade-off can be also seen in the insulation thickness. Increasing the insulation thickness leads to a better thermal insulation while also increasing the transport resistance of salt rejection. Therefore, in this work, we chose a macrochannel diameter of 2.5 mm and insulation thickness of 25.4 mm to ensure sufficient thermal localization and salt rejection.

With the optimized design parameters, the insulation foam, floating rings, copper plates, and macrochannels were machined using the waterjet (Supplementary Figure 2). Commercial black paint (245198, Rust-Oleum) was sprayed on the top surface of the insulation foam for the normal mode evaporator as well as on both sides of an aluminum plate for the contactless mode evaporator. Multiple copper plates were stacked together as the balancing weight to ensure the device floating on the water surface with a stable confined water layer. Different pieces were glued together with a waterproof polyurethane glue from Gorilla Inc. To fabricate the convection cover, two circular glass slides were cut by waterjet, wrapped by parafilm on the sidewall, and separated by a 5 mm air gap. The convection cover was used in configuration 2 and 3.

Supplementary Note 2: Cost analysis

A cost analysis is provided here for large-scale manufacturing of the device based on the current prices of raw materials. The device consists of floating structures, solar absorbing coating and a balancing weight. Mass-produced polyurethane foam can be purchased as the floating structures with ≈ 2.5 cm thickness, for up to \$2 m^{-3}.[1] The top surface can be painted with black marine coating for solar absorption, which is ≈ \$0.76 m^{-2}.[2] Finally, concrete offers a low-cost option as the balancing weight, at ≈ \$66-124 ton^{-1} depending on the locations,[3] which is equivalent to \$1.68-3.15 m^{-2} based on the weight needed for the floating structures. Overall, the total material cost of a wick-free self-floating confined water layer structure is ≈ \$2.5-3.9 m^{-2}.

Supplementary Note 3: COMSOL numerical simulation

The numerical simulation was conducted using finite element method in COMSOL Multiphysics v5.5, which coupled the transport of dilute species, laminar flow, and heat transfer into a time-dependent solver. The density of the brine solution is defined as $\rho(c,T) = \rho_{\mathrm{o}}(T) + \beta \cdot c$, where $\rho_{\mathrm{o}}(T)$ is the temperature dependent density of fresh water, c is the saline concentration and $\beta = 0.033\ \mathrm{kg\ mol^{-1}}$ is a proportionality constant. The density gradient is hence created by the temperature and concentration fields.

An evaporation heat flux was applied on the surface of the confined water layer as $q"_{\mathrm{evap}}(T) = h_{\mathrm{evap}}(T - T_{\mathrm{amb}})$, where $h_{\mathrm{evap}} = 53\ \mathrm{W\ m^{-2}\ K^{-1}}$ was calibrated based on the measured evaporation rate during the indoor experiments, T is the water-air interface temperature, and T_{amb} is the ambient temperature. Additionally, heat loss due to natural convection with $h_{\mathrm{natural}} = 5\ \mathrm{W\ m^{-2}\ K^{-1}}$ was applied on the water-air interface as well. To model the solar heating, a uniform heat flux $q"_{\mathrm{s}} = q"_{\mathrm{solar}}\alpha$ was applied on the top surface of the floating structure, where $q"_{\mathrm{solar}} = 1000\ \mathrm{W\ m^{-2}}$ is the incident solar flux and $\alpha = 0.953$ is the spectra averages solar absorptance (see Supplementary Figure. 3). To model the accumulated salt due to evaporation, a mass flux of NaCl was applied on the water-air interface as $J"_{\mathrm{evap}} = q"_{\mathrm{evap}} \cdot c/h_{\mathrm{fg}}/\rho$, where c, ρ, h_{fg} are the brine concentration, density, and latent heat at the water-air interface, respectively. The water-air interface was in a stress-free condition for the fluidic flow simulation. Other surfaces are modeled as no-flux boundaries and no-slip conditions for salt transport and fluidic flow, respectively.

The simulation domain was resolved by 385859 elements with tetrahedra as small as 0.3 mm. A refined mesh was applied to the boundary of confined water layer and macrochannels. The boundary conditions and meshing of the simulation domain are depicted in Supplementary Figure 4. A mesh dependent analysis was performed in Supplementary Figure 5 to confirm the numerical accuracy.

Supplementary Note 4: Outdoor characterization

An outdoor experiment was conducted on a sunny day (October 14, 2020) in East Setauket, New York, USA, including two identical experimental setups placed next to each other. The thermal insulation surrounding the test devices was constructed by polystyrene foam, with double-layer aluminum foil wrapped to avoid additional heating to the water reservoir. Each setup was placed on a digital balance to measure the evaporation rate during the test. To avoid the heating effect from the ground, both balances were placed on a 1-inch-thick polystyrene foam. The incident solar flux was measured by a pyranometer (SP-510-SS, Apogee), facing the same direction as the solar absorber. The experiment started at 10:30 (local time) and ended at 15:30 (local time). A Stevenson screen was placed at a similar height to the test devices to monitor the ambient temperature. Data collection was identical to the laboratory condition.

Supplementary Table 1: Comparison of solar-to-vapor conversion efficiency *η*, salt rejection capability, and cost of various solar evaporators

Solar evaporators	η (%)	Salinity (wt%)	Continuous testing time (h)	Salt crystallization	Cost
**This work**	**86**<br>**81**<br>**67**	**3.5**[a]<br>**20**[a]<br>**25**	**6 h**	**No**	**Low**
Self-assembled aluminum nanoparticles[4]	57	2.75	1 h	No	Medium
Femtosecond laser rendered metal panel[5]	67[b]	3.5	1 h	No	High
Fabric wick-polystyrene based solar evaporator[6]	55	3.5	0.55 h	No	Low
3D printed biomimetic solar evaporator[7]	96	25	9 h	Yes	High
Marangoni flow-driven salt rejection[8]	47[c]<br>130[d]	20	7 h	No	Medium
Filter paper-CNTs based solar evaporator[9]	81<br>71	3.5<br>13	600 h[e]	Yes[f]	Low
Electrospun Janus solar evaporator[10]	66	20	1 h	No	Medium
Natural wood[11]	75	20	100 h	No	Medium
Bimodal porous solar evaporator[12]	57	15	7 h	No	Medium
Janus wood[13]	82	20	8 h	Yes[g]	Medium
Water lily inspired solar evaporator[14]	79	10	8 h	No	Medium
MOF-derived porous carbon nanoflake arrays[15]	91	10	2.5 h	No	High

[a]7 cycles with 8 h per cycle; [b]clean water evaporation efficiency; [c]single-stage operation; [d]three-stage operation; [e]3.5 wt% saline water test; [f]salt crystallization at around 0.5 h for 3.5 wt% saline water test; [g]salt crystallization after 10-cycle operation.

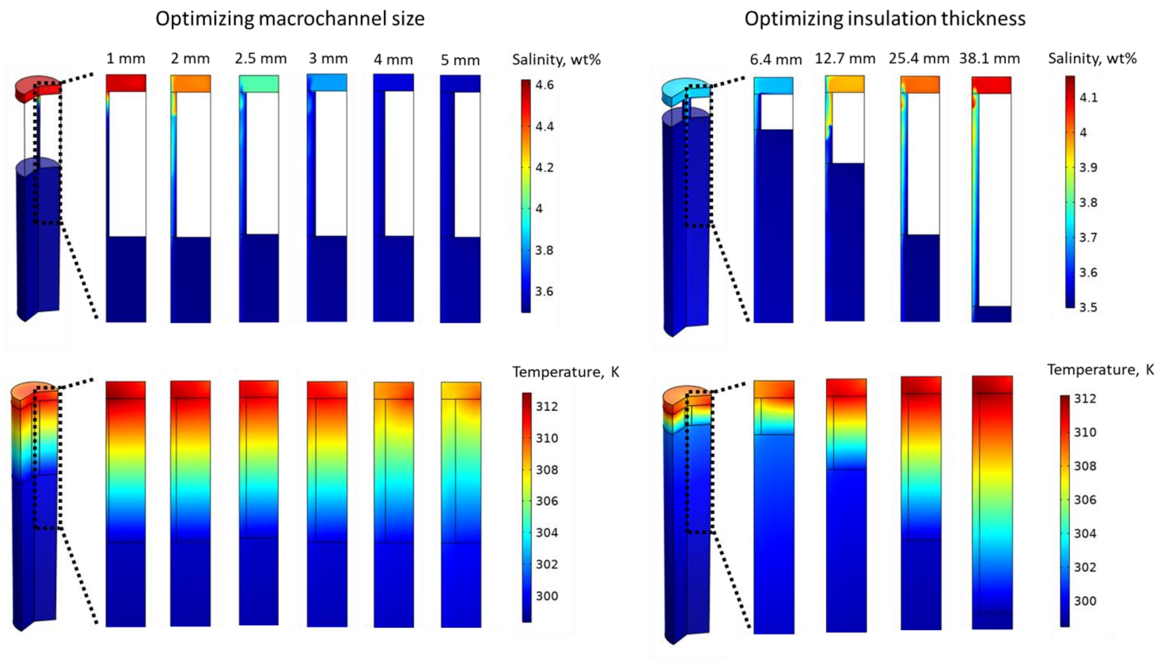

Supplementary Figure 1. Optimization of the macrochannel size and the insulation layer thickness to enable the simultaneous thermal localization and salt rejection. A single-channel unit with axial symmetry was simulated for the initial design optimization. Temperature and salinity profiles in the second hour are shown with different macrochannel sizes (1 mm to 5 mm) and insulation thicknesses (6.4 mm to 38.1 mm)

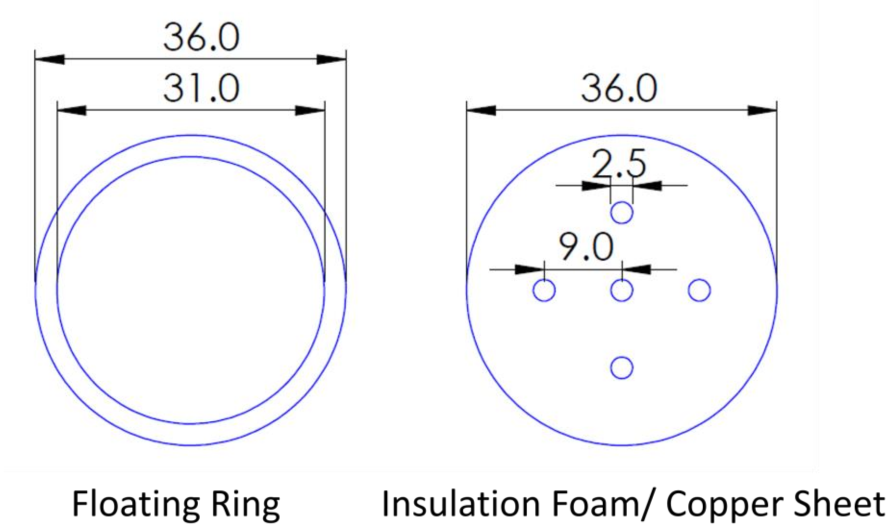

Supplementary Figure 2. Top view of the floating ring, insulation foam and copper sheets used for the waterjet machining. The dimensions were determined by the optimization process. Unit is in mm.

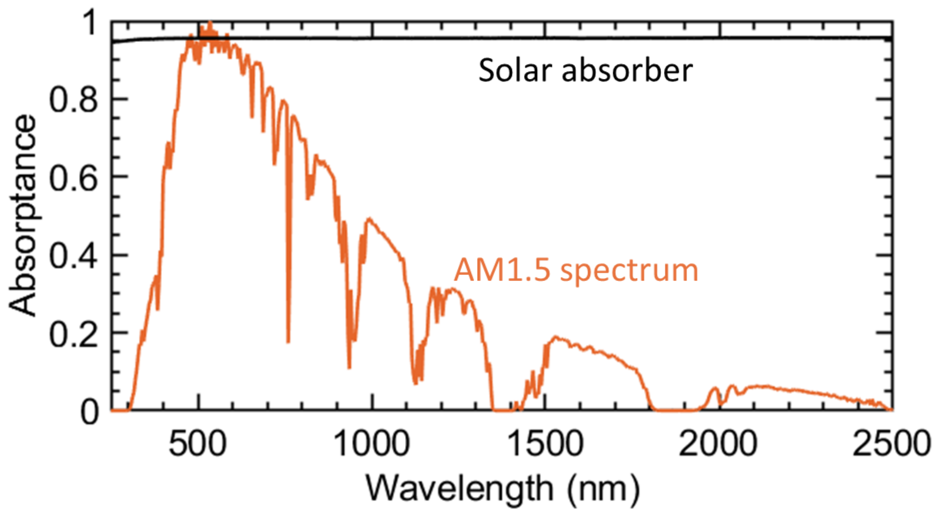

Supplementary Figure 3. Solar absorption characterization. The solar absorptance was characterized by a UV-vis-NIR spectrometer, represented by the black curve. Commercial black painted layer was used as the solar absorber. The spectra averaged solar absorptance was 95.3±0.5%, which was calculated based on the AM1.5 solar spectrum (orange curve). The uncertainty was determined by measurements on multiple samples.

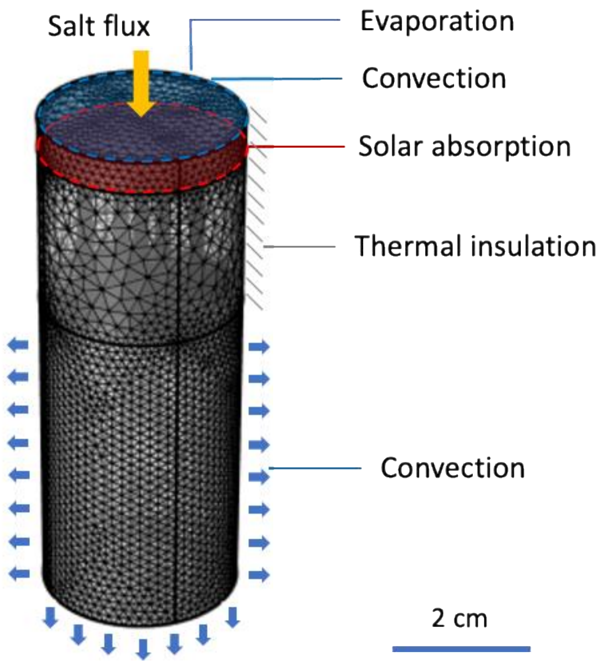

Supplementary Figure 4. Simulation domain with boundary conditions and meshing elements. A total number of 385859 elements were created for meshing, with tetrahedra elements as small as 0.3 mm. A time-dependent solver was applied to couple the heat and salt transport with the fluidic flow.

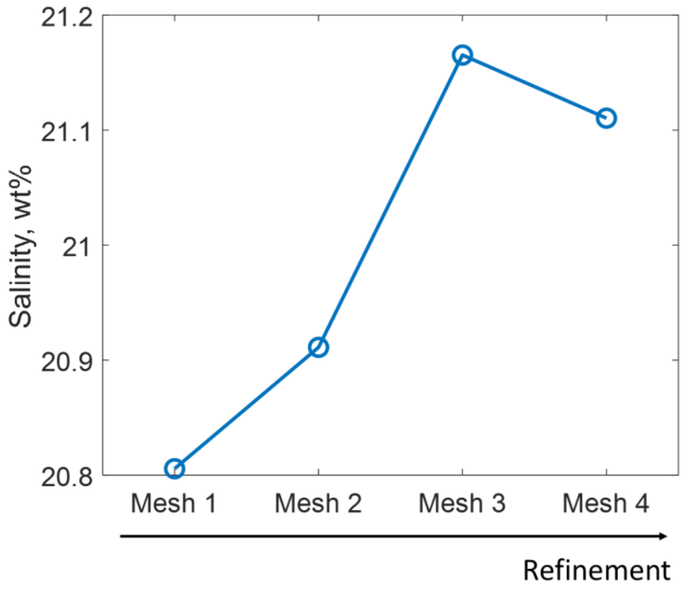

Supplementary Figure 5. Mesh dependent analysis. Confined water layer salinity in the first half hour was simulated using different mesh refinements. Mesh 1-4 represent different meshing configurations, which include 64741, 163446, 385859, and 1218068 elements, respectively. Simulation results gradually converged with the increase of refinement. Mesh 3 was chosen for the numerical simulation to ensure the computational accuracy and efficiency.

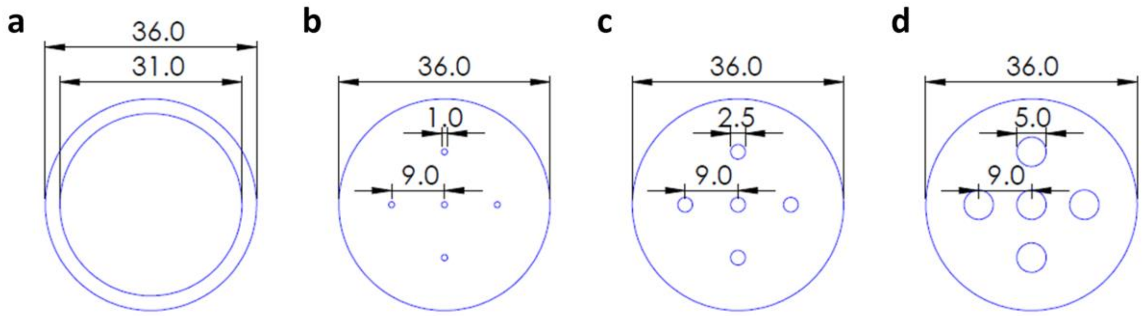

Supplementary Figure 6. Top view of the (a) floating ring, insulation foams with (b) 1 mm, (c) 2.5 mm, and (d) 5 mm macrochannel diameters used for experimental characterizations. Unit is in mm.

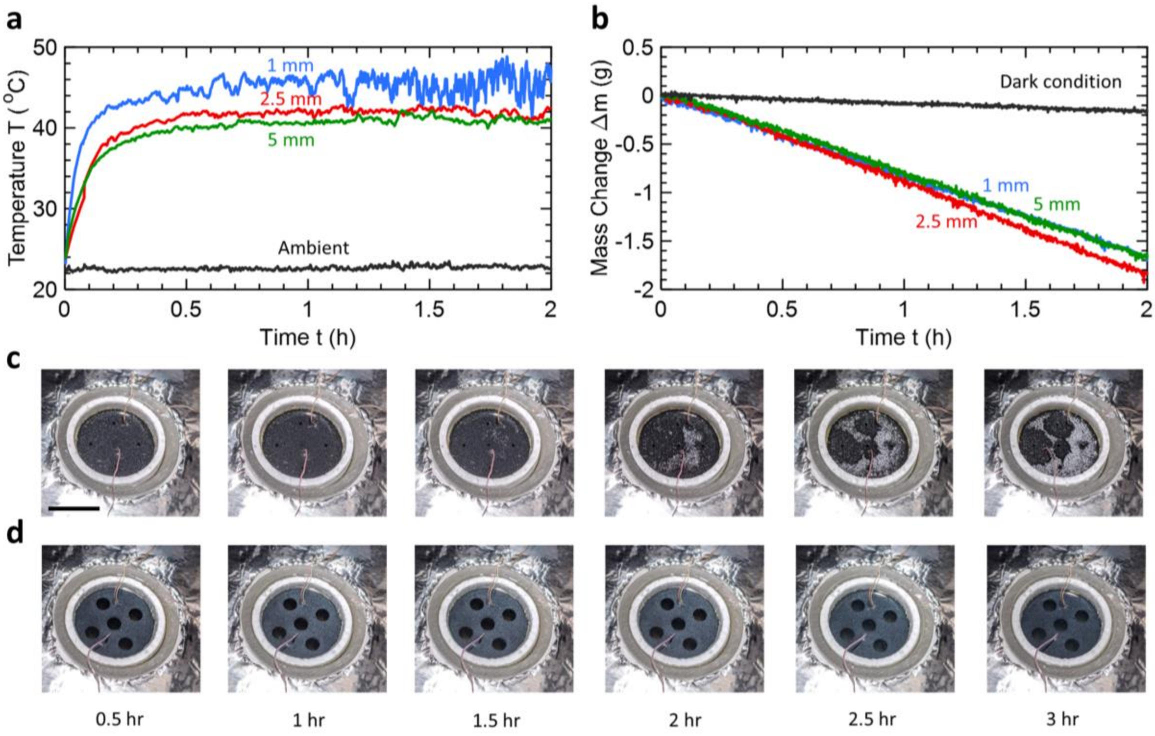

Supplementary Figure 7. Solar evaporation of 20 wt% brine under one sun illumination. Three confined water layer structures with 1 mm, 2.5 mm, and 5 mm diameter macrochannels were tested (see Supplementary Figure 6 for detailed dimensions). (a) Temperature response of the confined water layer with different macrochannel diameters. The steady state temperature decreases with macrochannel diameters due to the increased heat loss through the floating insulation, which validates our theoretical prediction in Fig. 3a of the main text. (b) Corresponding mass change of the evaporation setup as a function of time. Confined water layer structure with 2.5 mm diameter macrochannels shows the highest evaporation rate, which was chosen for our final design. The reduced evaporation rate of the 1 mm diameter macrochannel design is attributed to the salt crystallization and increased convective heat loss due to the elevated water layer temperature, whereas the reduced evaporation rate of the 5 mm diameter macrochannel design is induced by the increased conductive heat loss through macrochannels. Time-lapse images of the confined water layer structure with (c) 1 mm and (d) 5 mm diameter macrochannels during a 3-hour continuous solar evaporation. Salt crystallization was observed on the 1 mm diameter macrochannel design after the second hour, because natural convection enhanced salt rejection cannot be initiated with small macrochannel diameters. Scalebar: 2 cm.

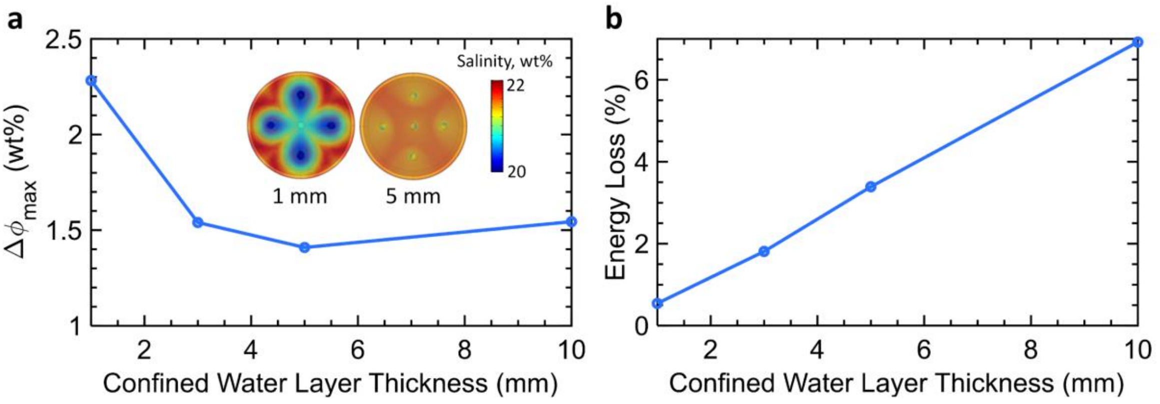

Supplementary Figure 8. Effects of the confined water layer thickness on (a) salt transport and (b) heat loss predicted by simulations. Solar evaporation of 20 wt% brine under one sun illumination was simulated. The macrochannel diameter is 2.5 mm. (a) Nonuniformity of salt concentration $\Delta\phi_{max}$ as a function of the confined water layer thickness at the transition point to the quasi-steady state (*i.e.*, ≈ 0.5 hours after the simulation started). $\Delta\phi_{max}$ is the maximum value of salt concentration difference in the confined water layer, which is defined as the difference between the highest salt concentration and the lowest salt concentration. Insets: top view of salt concentration profiles with 1 mm and 5 mm thick confined water layers. Nonuniform salt concentration is undesirable because salt crystallization will first occur at the position with the highest salt concentration. $\Delta\phi_{max}$ first rapidly decreases and then slightly increases with the increase of the confined water layer thickness, leading to a minimum point approximately at 5 mm, which was selected for our design. The large nonuniformity of salt concentration occurs at a low confined water layer thickness (*e.g.*, 1 mm), because the resistance of salt transport along the confined water layer (*i.e.*, the lateral transport) is inversely proportional to its thickness, resulting in a low salt concentration near the macrochannel with a high salt concentration close to the external floating ring (see the inset of (a)). The slight increase in salt concentration nonuniformity at the large thickness (*e.g.*, 10 mm) is attributed to the increased resistance of salt transport crossing the confined water layer (*i.e.*, the vertical transport). (b) Heat loss through the sidewall of a floating ring as a function of the confined water thickness. Heat loss increases linearly due to the increase of sidewall area.

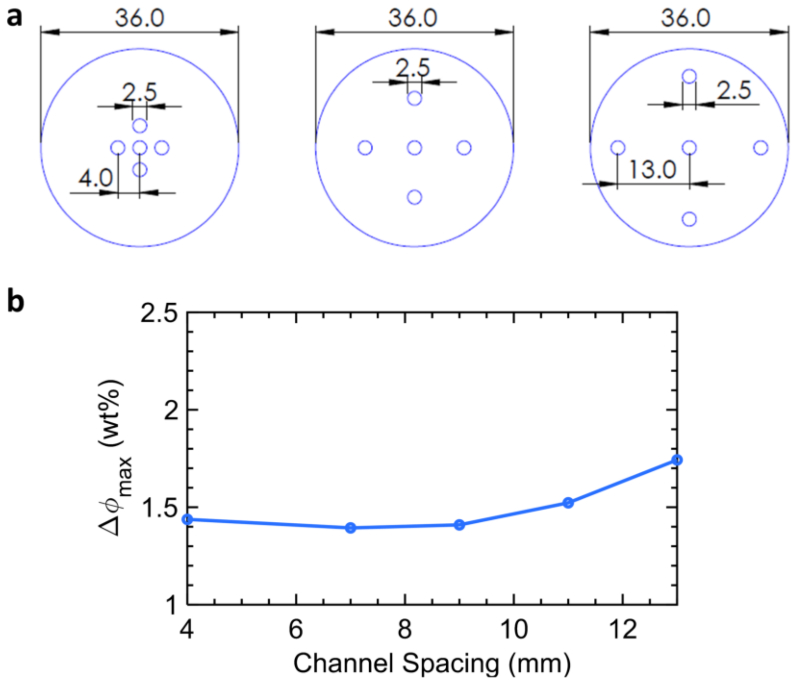

Supplementary Figure 9. Effects of macrochannel spacing on the nonuniformity of salt concentration predicted by simulations. Solar evaporation of 20 wt% brine under one sun illumination was simulated. The macrochannel diameter is 2.5 mm and the confined water layer thickness is 5 mm. (a) Top view of three representative insulation foams with 4 mm, 9 mm, and 13 mm macrochannel spacing used for simulations. Unit is in mm. (b) Nonuniformity of salt concentration as a function of macrochannel spacing. In general, $\Delta\phi_{max}$ weakly depends on the macrochannel spacing, but too large of a macrochannel spacing will lead to an increase of salt concentration nonuniformity. 9 mm spacing was chosen for our design to achieve the optimal macrochannel arrangement.

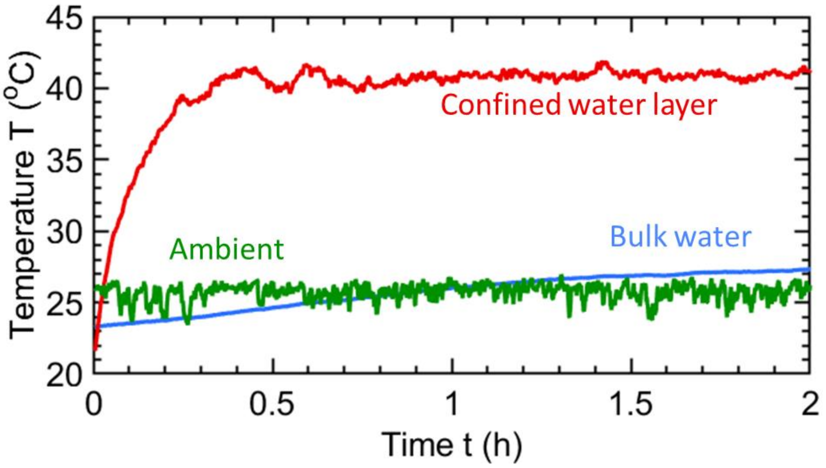

Supplementary Figure 10. Temperature response of configuration 2. By adding a convection cover, configuration 2 can further enhance the thermal localization by reducing the convective loss while maintaining similar energy efficiency and evaporation rate.

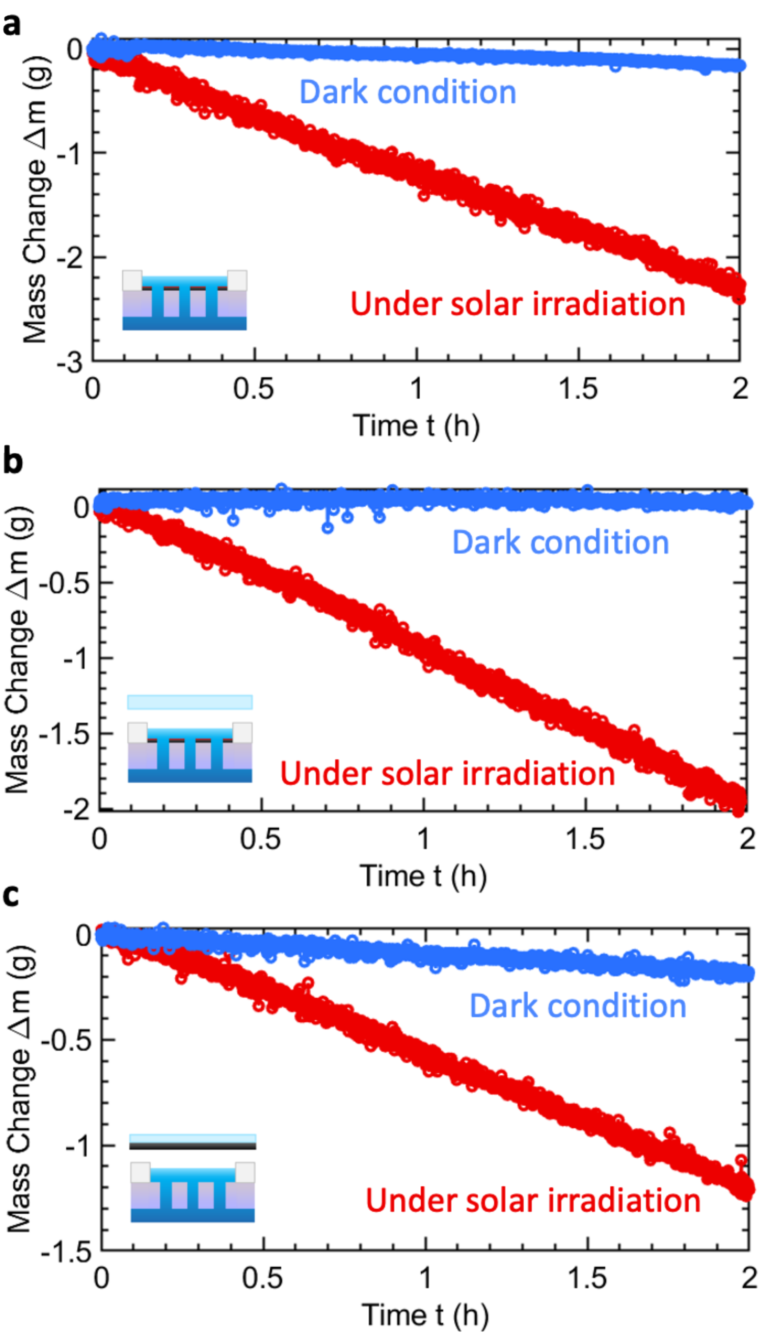

Supplementary Figure 11. Evaporation mass change curves under solar illumination and in the dark conditions. a, Configuration 1: normal mode evaporator without convection cover. **b,** Configuration 2: normal mode evaporator with convection cover. **c,** Configuration 3: contactless mode evaporator. The dark evaporation rates were 0.095 g h^{-1}, 0.017 g h^{-1}, and 0.076 g h^{-1} for configurations 1-3, respectively. Solar-to-vapor conversion efficiency was determined by excluding the dark evaporation rate from the total evaporation rate.

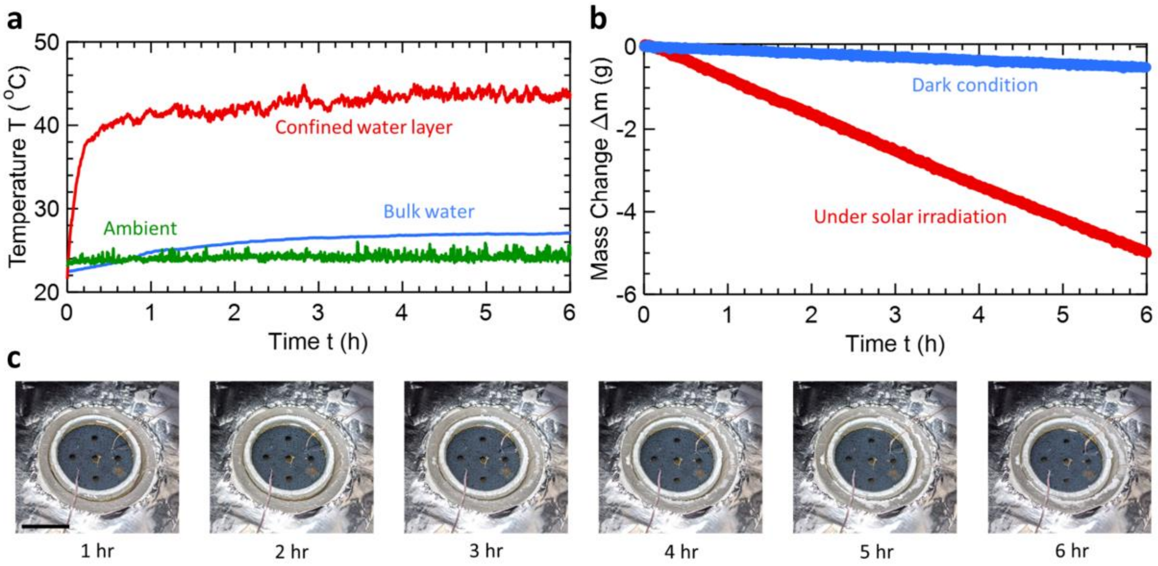

Supplementary Figure 12. 6-hour continuous solar evaporation of 25 wt% brine under one sun illumination. (a) Temperature response of the confined water layer. (b) Corresponding mass change of the evaporation setup as a function of time. (c) Time-lapse images of the confined water layer structure during the 6-hour continuous solar evaporation. Scalebar: 2 cm. The brine concentration (25 wt%) is approaching to the saturation point (26 wt%) at room temperature. No salt crystallization was observed during the 6-hour continuous solar evaporation, demonstrating the superior salt rejecting performance of the confined water layer structure. The corresponding solar-to-vapor conversion efficiency is 67%.

References

1. Polyurethane Foam Prices. Available at: https://www.alibaba.com/product-detail/Hard-Surface-custom-high-quality-PVC_60456172600.html.
2. Black Marine Coating Prices. Available at: https://www.alibaba.com/product-detail/High-build-Epoxy-Coal-Tar-Pitch_62538348089.html.
3. Concrete Prices. Available at: https://www.sci99.com/monitor-94896214-0.html; https://www.statista.co%0Am/statistics/219339/us-prices-of-cement/ %0A.
4. Zhou, L. *et al.* 3D self-assembly of aluminium nanoparticles for plasmon-enhanced solar desalination. *Nat. Photonics* **10**, 393–398 (2016).
5. Singh, S. C. *et al.* Solar-trackable super-wicking black metal panel for photothermal water sanitation. *Nat. Sustain.* **3**, 938–946 (2020).
6. Ni, G. *et al.* A salt-rejecting floating solar still for low-cost desalination. *Energy Environ. Sci.* **11**, 1510–1519 (2018).
7. Wu, L. *et al.* Highly efficient three-dimensional solar evaporator for high salinity desalination by localized crystallization. *Nat. Commun.* **11**, 1–12 (2020).
8. Morciano, M., Fasano, M., Boriskina, S. V., Chiavazzo, E. & Asinari, P. Solar passive distiller with high productivity and Marangoni effect-driven salt rejection. *Energy Environ. Sci.* **13**, 3646–3655 (2020).
9. Xia, Y. *et al.* Spatially isolating salt crystallisation from water evaporation for continuous solar steam generation and salt harvesting. *Energy Environ. Sci.* **12**, 1840–1847 (2019).
10. Xu, W. *et al.* Flexible and Salt Resistant Janus Absorbers by Electrospinning for Stable and Efficient Solar Desalination. *Adv. Energy Mater.* **8**, (2018).
11. Kuang, Y. *et al.* A High-Performance Self-Regenerating Solar Evaporator for Continuous Water Desalination. *Adv. Mater.* **31**, (2019).
12. He, S. *et al.* Nature-inspired salt resistant bimodal porous solar evaporator for efficient and stable water desalination. *Energy Environ. Sci.* **12**, 1558–1567 (2019).
13. Chen, X. *et al.* Sustainable off-grid desalination of hypersaline waters using Janus wood evaporators. *Energy Environ. Sci.* **14**, 5347–5357 (2021).

14. Xu, N. *et al.* A water lily-inspired hierarchical design for stable and efficient solar evaporation of high-salinity brine. *Sci. Adv* **5**, eaaw7013 (2019).

15. Zhang, Y. *et al.* Manipulating unidirectional fluid transportation to drive sustainable solar water extraction and brine-drenching induced energy generation. *Energy Environ. Sci.* **13**, 4891–4902 (2020).
