# Supplementary material for: Highly efficient and salt rejecting solar evaporation via a wick-free confined water layer
Source: Nat Commun. 2022 Feb 14;13:849. doi: 10.1038/s41467-022-28457-8 (PMC8844429; doi:10.1038/s41467-022-28457-8)
Supplement: Supplementary file 3 — Description of Additional Supplementary Files [file 41467_2022_28457_MOESM3_ESM.pdf]

Description of Additional Supplementary Files

File Name: Supplementary Movie
Description: **Dripping tests of 1 mL food dye colored deionized (DI) water (left) and 20 wt% brine onto the confined water layer.** Water reservoir initially contained DI water only. The dripping process was completed at the sixth second. The zeroth second for the liquid transport visualization shown in Fig. 3c,d of main text corresponds to the sixth second of this video.
